# Nutrition in Spondyloarthritis and Related Immune-Mediated Disorders

**DOI:** 10.3390/nu14061278

**Published:** 2022-03-17

**Authors:** Stefan Lucian Popa, Dinu Iuliu Dumitrascu, Vlad Dumitru Brata, Traian Adrian Duse, Maria Delia Florea, Abdulrahman Ismaiel, Laura Mirela Muntean, Simona Grad

**Affiliations:** 1 2nd Medical Department, “Iuliu Hatieganu” University of Medicine and Pharmacy, 400000 Cluj-Napoca, Romania; popa.stefan@umfcluj.ro (S.L.P.); abdulrahman.ismaiel@yahoo.com (A.I.); costinsimona_m@yahoo.com (S.G.); 2 Department of Anatomy, “Iuliu Hatieganu” University of Medicine and Pharmacy, 400006 Cluj-Napoca, Romania; 3 Faculty of Medicine, “Iuliu Hatieganu” University of Medicine and Pharmacy, 400000 Cluj-Napoca, Romania; brata_vlad@yahoo.com (V.D.B.); adrianduse@yahoo.com (T.A.D.); floreamaria98@gmail.com (M.D.F.); 4 Rheumatology Department, “Iuliu Hațieganu” University of Medicine and Pharmacy Cluj, 400012 Cluj-Napoca, Romania; lmuntean13@yahoo.com

**Keywords:** nutrition, spondyloarthritis, HLA-B27, ankylosing spondylitis, psoriatic arthritis, inflammatory bowel disease, therapy

## Abstract

Recent research on the pathogenesis of spondyloarthritis and related immune-mediated diseases associated with human leukocyte antigen class I molecule B27 (HLA-B27) has led to significant progress in terms of management and prognosis, with multiple treatments being constantly evaluated and implemented. Correlations between the genetic background of spondyloarthritis and inflammatory bowel diseases and the inflammatory processes involving gut microbiota have been established. This knowledge has allowed progress in pharmacological therapy. The role of diet in the pathogenesis and treatment of diseases pertaining to the HLA-B27 spectrum is of great significance, considering possible future applications in individualized medicine. Diet impacts the composition of gut microbiota, representing a substrate for the synthesis of metabolites affecting the mucosal immune system. Certain pro-inflammatory mediators, such as emulsifiers and microparticles, induce a more profound cytokine response, promoting inflammation. Numerous diets, including the low-starch diet, the Mediterranean diet, diets with low contents of fermentable oligosaccharides, disaccharides, monosaccharides and polyols (low-FODMAP diets), gluten-free diets and fasting, have been analysed and correlated with patients’ symptomatology and dietary adherence. The aim of this review is to provide an extensive perspective on the diets available to patients with spondyloarthritis and related immune-mediated disorders.

## 1. Introduction

Spondyloarthritis (SpA) is a heterogenous group of interconnected immune-mediated inflammatory diseases. These diseases have common features, including inflammation of sacroiliac joints and spine, peripheral arthritis, enthesitis, dactylitis and extra-articular manifestations, such as uveitis, psoriasis and inflammatory bowel disease (IBD) [1,2]. According to the main clinical features, recent classification criteria developed by the Assessment of SpondyloArthritis International Society (ASAS) separate axial and peripheral SpA. Axial SpA, comprising ankylosing spondylitis, is characterized by radiographic changes in the sacroiliac joints and non-radiographic axSpA (nr-axSpA), without radiographic sacroiliitis [1]. Peripheral SpA encompasses a broad spectrum of different but interconnected diseases, including psoriatic arthritis, reactive arthritis and arthritis associated with IBD (enteropathic arthritis) [2]. Regardless of the subtype, the most common clinical manifestations are inflammatory back pain, stiffness and swelling of the spine and/or peripheral joints, enthesitis, dactylitis, as well as extra-articular manifestations, such as uveitis, psoriasis and IBD [1,2]. 

Apart from similar presentation, SpA diseases share a common genetic background, being strongly associated with HLA-B27 [2,3]. Even though the frequency of the allele is less than 10% in the general population, more than 95% of AS patients are HLA-B27-positive [4]. In contrast to the high prevalence in SpA patients with ankylosing spondylitis, HLA-B27 is positive in less than 50% of patients with PsA and IBD [5,6,7,8]. HLA-B27 is a heterodimer consisting of two covalently linked polypeptide chains, α and β2-microglobulin. While the β2 chain is encoded by a non-HLA gene, the alpha chain, encoded by the B locus of the MHC gene on chromosome 6 (HLA-B), is polymorphic in nature and subsequently binds an array of specific antigenic peptides. The complex, together with the surface CD8 antigen is then presented to cytotoxic T lymphocytes [3,9]. This ability to recognize certain antigens is thought to be the reason behind the association with the disease spectrum of SpA.

Since the discovery of the association with SpA in the 1970s, four major theories explaining the role of HLA-B27 in the molecular pathogenesis of SpA have been developed, although the exact mechanisms are still unknown [10]. These four predominant hypotheses revolve around arthritogenic peptides, cellular response to unfolded proteins, cell-surface HLA-B27 homodimer formation and ERAP1 and ERAP2 polymorphisms [1,9]. A more recent theory explaining the pathogenesis of SpA involves the relationship between the gut microbiome, intestinal inflammation and autoimmune diseases [11]. The gut microbiome plays a key role in the host’s innate intestinal immune response and alterations to its composition and dysbiosis are linked to autoimmune and inflammatory diseases (including those in the spectrum of SpA) through mechanisms such as increased epithelial permeability, activation of the immune response and molecular mimicry [3,11,12]. A wide array of studies has shown that SpA patients have distinct compositions of microbiota compared to controls and more than 60% have microscopic evidence of intestinal inflammation [13]. Furthermore, this hypothesis is supported by significant overlap between SpA and IBD. For example, up to 50% of IBD patients develop SpA, while 7% of AS patients also have IBD [14].

In spite of the significant implication of genetic background in the pathogenesis, numerous lifestyle factors, including diet, play an essential role in the onset, natural evolution and prognosis of these disorders. Several diets—their characteristics and specific impacts on spondyloarthritis and related immune-mediated disorders—have been analysed, such as a “low-starch diet”, consisting of low quantities of carbohydrates, such as bread, potatoes, biscuits, pasta and cereals, and increased quantities of red and white meat, fish, vegetables and fruits in order to compensate for the loss of calories [15]. Additionally, the effect of fasting or the exclusion of certain foods (dairy or products rich in flour) has also been assessed [16,17,18].

The Mediterranean diet (Md) is characterized by high intakes of fruit and vegetables, whole grains, polyunsaturated fats, such as olive oil, and proteins from fish, legumes and nuts, and is adopted as a part of treatment for many diseases due to its anti-inflammatory properties [19]. In comparison, the “westernized diet” represents a hypercaloric type of diet, richer in saturated fats, sugars, refined carbohydrates, emulsifiers and animal proteins, with concomitantly smaller quantities of fresh vegetables, fruits and fiber [20].

FODMAPs are molecules that are poorly absorbed by the intestine and fermented by the bacteria making up the colon microbiome and as such may be responsible for gastrointestinal symptoms in IBD via excessive gas production and intraluminal osmotic effect [21,22]. Thus, diets low in FODMAPs have been analysed in correlation with diseases associated with HLA-B27.

Managing all these intricate disorders is demanding and frequently requires a skillfully coordinated approach involving different specialists. For this reason, a multidisciplinary team from a range of different professions (including gastroenterologists, rheumatologists, ophthalmologists, psychiatrists, psychologists, dermatologists, cardiologists, radiologists, physiotherapists, dietitians, pharmacists and nurses) provide the best support for patients with HLA-B27-positive disorders and their family members [23]. 

**Figure 1:**
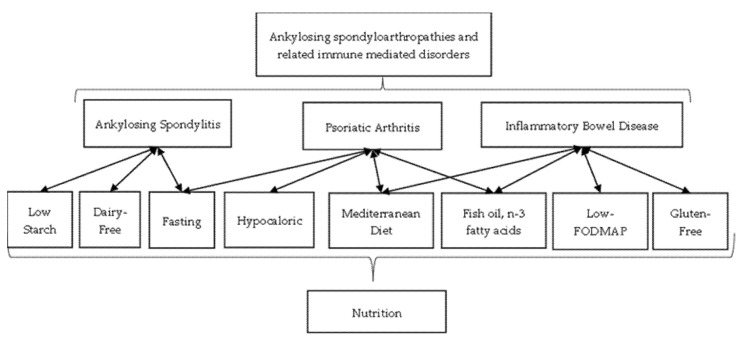
Nutrition in spondyloarthritis and related immune-mediated disorders.

Since several studies have analyzed the effect of diet in the majority of these inflammatory disorders, the aim of this review is to find out whether one particular diet may be efficient as a non-pharmacological therapy in all spondyloarthritis and related immune-mediated disorders. The association between nutrition and spondyloarthritis and related immune-mediated disorders is illustrated below in Figure 1.

## 2. Ankylosing Spondylitis

Recent data suggest that HLA-B27 may predispose to AS by altering the intestinal microbiome [1,5,24,25]. Evidence of a possible link between microbiota and AS has emerged from animal models, with evidence suggesting that HLA-B27 transgenic rats do not develop arthritis and colitis under germ-free conditions, but, when exposed to commensal bacteria, develop arthritis and colitis, respectively [26]. Studies in humans revealed that patients with AS exhibit significant differences in the constituents of their intestinal microbiota when compared to healthy controls [27,28,29]. Recent data suggested that a common alteration in microbiota of AS patients is decreased microbial diversity [27,28,29]. Moreover, several specific bacteria were found to be significantly correlated with disease activity [25,29,30,31]. Diet is an important modifiable factor which modulates the composition, diversity and stability of intestinal microbiota; it is assumed that nutritional intervention may restore microbiota richness in inflammatory-mediated diseases [32].

In addition, the role of a previous bacterial infection in the complex pathophysiology of AS was investigated. For example, some studies have analysed the correlation between *Klebsiella pneumoniae* and AS and the ways in which certain strains of this bacteria might impact the disease [33,34]. Ebringer et al. prelevated faecal cultures from 163 patients with AS in order to assess the relationship between *Klebsiella pneumoniae* and disease activity, concluding that a higher number of bacterial colonies was associated with increased disease activity [33]. Thaiss et al. also found evidence that patients with active AS have higher levels of IgA, particularly against *Klebsiella pneumoniae*, further pointing to a correlation between the two entities [35]. Additionally, studies of various bacteria have concluded that starch represents an important source of energy for the gut microbiome, with diets high in starch intake leading to increased colonisation of the human gut with *Klebsiella pneumoniae* [36,37]. The main diets investigated in relation to AS are presented in Table 1.

The research regarding the impact of diet in modulating disease activity and symptoms in AS remains limited. A small number of studies have examined the elimination of various nutrients from the diet in the management of AS. In a longitudinal observational study, Appelboom et al. studied the effects of a dairy-free diet on 25 patients with AS [38]. Self-reported beneficial effects were obtained in 52% of patients after six weeks of follow-up, out of which 62% could discontinue their treatment with nonsteroidal anti-inflammatory drugs. Only 24% of studied patients were adherent to this diet after 2 years [38]. When it comes to the relationship between the impact of the diet and the HLA-B27-positive/negative status of the included patients, the authors concluded that the effect of the said diet was independent of the presence or absence of HLA-B27 [38].

The hypothesis that a diet consisting of a high starch intake might be associated with increased disease activity in AS patients was tested by Ebringer and Wilson by implementing a low-starch diet for a period of nine months with 36 patients positively diagnosed with AS [15]. IgA and erythrocyte sedimentation rate (ESR) were monitored both before the implementation of the low-starch diet and after and revealed that, before the diet, no reduction in ESR or IgA occurred. However, IgA and ESR significantly decreased over the nine-month period following the implementation of this type of diet [15]. The so-called “low-starch diet” consisted of low quantities of carbohydrates, such as bread, potatoes, biscuits, pasta and cereals, and increased quantities of red and white meat, fish, vegetables and fruits to compensate for the loss of calories [15].

Sundström et al. conducted a study in order to assess the relationship between diet, disease activity and gastrointestinal symptoms in patients diagnosed with AS [39]. Data from 111 participants were analysed based on questionnaires regarding socio-demographic aspects, dietary habits, including dietary supplements, physical activity, the use of NSAIDs and gastrointestinal symptoms. Moreover, disease activity and functional capacity were also measured using the Swedish versions of the Bath Ankylosing Spondylitis Disease Activity Index (BASDAI) and the Bath Ankylosing Spondylitis Functional Index (BASFI) [39]. The study concluded that 27% of the participants reported gastrointestinal symptoms when consuming certain types of foods, such as dairy products or foods rich in flour [39]. Although the study did not find any correlation between diet and disease activity, the high percentage of patients reporting gastrointestinal symptoms when consuming certain food products containing starch further demonstrates the impact such products have on the gut microbiome and, implicitly, on the general wellbeing of patients [39].

Regarding certain foods that have been incriminated in the aggravation of symptoms in patients with AS, the effect of fasting on the evolution of the disease was also investigated. Several studies have analysed the impact that fasting has on the progression or remission of symptoms in patients suffering from various rheumatic diseases, including AS [17,40]. A study by Haugen et al. included 41 patients diagnosed with AS who answered questionnaires provided regarding different types of diet and fasting [40]. When it came to fasting for an average period of 7–10 days, more than half of the respondents reported less pain, less stiffness and less joint-swelling, although the results were similar among all rheumatic diseases and not specific to patients suffering from AS [40]. Moreover, the patients were interviewed regarding diet treatments undergone and their effects on the disease, with 47% reporting less pain, 46% less stiffness and 36% reduced joint swelling, without any differences regarding the type of rheumatic disease incriminated [17]. No particular diet has been analysed such that its making a significant improvement to the symptomatology of patients with AS in comparison with other diets can be concluded [40].

Recently, Macfarlane et al. published a systematic review of the relationship between diet and AS [41]. Only 16 clinical trials, the majority small case–control studies, with the quality of scientific evidence either low or very low, were included. Overall minimal benefits were seen in some patients, with no clear influence on objective markers of disease activity [41]. These data highlighted the uncertainty of responses and important methodological limitations in the studies reviewed.

Despite limited data, Feldtkeller et al. proposed a core set of recommendations for AS patients concerning behaviours and environmental adaptations, including diet [42]. Patients were recommended to include in their diet more plant foods, vegetables and fruits, as well as fish meals, and to reduce meat consumption. In addition, for osteoporosis prevention, they were advised to have an optimal intake of vitamin D and calcium [42]. Further prospective longitudinal studies addressing the role of nutrition could lead to strategies that improve quality of care in patients with AS.

## 3. Psoriatic Arthritis

Psoriatic arthritis (PsA) is an inflammatory arthropathy affecting up to 30% of psoriasis patients [43]. The prevalence of the disease is equal for males and females, ranging between 0.06–0.25% in the US and 0.05–0.21% in Europe, with discrepancies between the studies deriving from the different definitions of psoriatic arthritis [44,45]. However, recent findings suggest the prevalence may be higher [44]. Psoriasis precedes the manifestations of psoriatic arthritis by ten years in most cases, although the symptoms may occur simultaneously, or the arthritis can present first [44]. Psoriatic arthritis can manifest itself in five clinical subtypes: distal, oligoarticular, polyarticular, axial and arthritis mutilans [44]. Pathogenetic studies show that different HLA-B27 alleles determine susceptibility to and the phenotype of the disease [46]. Nonetheless, the disease drastically affects quality of life, being compared to the burdens of axial spondyloarthritis and rheumatoid arthritis [47,48]. Furthermore, we present in Table 2 the influence that different diets have in the manifestations of psoriatic arthritis.

The impact of Md on the pathology of PsA was evaluated in a cross-sectional observational study conducted by Caso et al. [19]. The cohort consisted of 211 consecutive PsA patients from five rheumatology units in Italy, monitored from January to May 2019. The patients included in the study were both males and females, over the age of 18, fulfilling the Classification Criteria for Psoriatic Arthritis (CASPAR) [19]. The adherence to the diet was measured by PREMED (a validated 14-item questionnaire) and PsA activity was quantified using DAPSA (Disease Activity Index for Psoriatic Arthritis). Additionally, patients suffering from endocrinopathies and patients treated with corticosteroids or progestines in the last 6 months were excluded from the cohort. The study closely follows the adherence of the subjects to Md and the impact of the diet on the activity of the disease. The results show that low adherence to Md is associated with high PsA activity. Although more studies are needed to assess the impact of this diet on the severity of the disease, the study shows that patients may benefit from it’s anti-inflammatory properties [19].

Furthermore, it is a well-known fact that the developed countries tend to adopt a “westernized diet”. This pattern of consumption has led to a higher prevalence of obesity, diabetes, cardiovascular diseases and metabolic syndrome. Obesity is often associated with increased disease activity in PsA and a lower response to treatment [20]. One interventional study conducted by Klingberg et al. concluded that the 41 patients suffering from PsA and obesity benefited from the very low-energy diet (640 kcal/day), resulting in a decreased disease activity in joints, enthuses and skin [20]. The patients suffering from PsA (CASPAR criteria) and obesity (BMI ≥ 33 kg/m^2^) followed the very low energy diet over a period of 12 weeks with minor side effects, such as nausea, headaches and obstipation. After the 12 weeks, patients were required to follow a personalized energy-restricted diet. The study shows significant reductions in disease activity at the 6 months check-up and a larger weight loss was associated with an improvement in a dose–response manner [20]. Moreover, Di Minno et al. [49] evaluated the impact of weight loss on MDA (minimal disease activity) in overweight or obese patients suffering from PsA and at the beginning of treatment with TNFα (tumour necrosis factor α) blockers. Out of the 126 patients included in the study, 63 followed a hypocaloric diet (<1500 kcal/day), while the other 63 had a free-managed diet. Patients were closely monitored over a 30-month period, including metabolic measurements, monthly rheumatological evaluation and a full examination once every 6 months. The study concluded that, regardless of the diet followed, a ≥5% weight loss is the major predictor of MDA achievement [49]. 

The limited data on the impact of a gluten-free diet in PsA patients cannot determine whether this change in the patient’s diet can reduce the severity of PsA [53,54,55]. On the other hand, studies concluded that vitamin D supplementation positively alleviated the severity of the disease using uncontrolled groups [56,57]. A randomized controlled trial concluded that a daily combination of 48 μg selenium aspartate, 50 mg coenzymeQ10 (ubiquinone acetate) and 50mg vitamin E (α-tocopherol) has a positive outcome on PsA patients, in addition to conventional therapy [50]. Another randomized controlled trial conducted by Kristensen et al. concluded that patients diagnosed with PsA had a reduced disease activity and use of NSAID and paracetamol when following a diet rich in n-3 polyunsaturated fatty acids, commonly found in fish oil [51]. There are no conclusive data on the impact of vitamin B12, selenium or micronutrient combinations on PsA activity. 

In addition to the extensive research conducted on various diets and dietary supplements, fasting has also been analysed regarding its implication in the severity of PsA. Fernando et al. conducted a systematic review and meta-analysis to determine the effects of Ramadan practice on weight loss and body composition in otherwise healthy adults, concluding that the intermittent fasting had a positive effect that resulted in weight loss and a lower fat percentage in overweight or obese patients [58]. Adawi et al. designed a study that overlooks the impact of Ramadan fasting on PsA activity and associated pathologies, such as enthesitis and dactylitis [52]. The study included 37 PsA patients from three medical centers who were in the maintenance phase of their medication, had no disease severity changes in the last two clinical visits and were not positive for hepatitis B or C or HIV. Although the study found no difference in weight loss, Ramadan practice had beneficial effects on disease activity scores, DAPSA (Disease Activity Index for Psoriatic Arthritis) and BASDAI (Bath Ankylosing Spondylitis Disease Activity Index) [52]. Due to the small number of participants in the study and the short period of intermittent fasting (one month), larger studies are needed to confirm the findings.

Considering the analysed studies, healthy diets, such as the Mediterranean diet, the intermittent fasting practiced during Ramadan, a low-calorie intake diet and vitamin D supplementation can alleviate the severity of the disease and reduce the oxidative stressors that exacerbate the disease.

## 4. Inflammatory Bowel Disease

IBDs, with their two main forms, Crohn’s disease (CD) and ulcerative colitis (UC), are chronic immune-mediated diseases characterized by repeated episodes of remission and relapse [59]. Their incidence is growing worldwide, especially in westernized countries, with the highest incidences reported in Northern Europe, North America and the United Kingdom. Although a rare occurrence in the past, the incidence of inflammatory bowel diseases seems to be increasing in developing countries as they become more industrialized [60,61,62]. This rise in traditionally low-incidence areas has led researchers to believe that environmental factors commonly associated with the so-called “westernized lifestyle” play a key role in the development of IBD [63,64].

The precise pathogenesis of IBD remains a mystery, despite extensive research in the area, but the current belief is that the development of the disease implies an abnormal gut immune response stimulated by a modified microbiome in genetically predisposed people [59,65]. Although the frequency of HLA-B27 in IBD patients is not significantly different from that in the general population, patients who suffer from both IBD and ankylosing spondylitis (AS) have been shown to have a significantly higher HLA-B27 association when compared to IBD patients without AS [66,67,68].

One factor that has seen considerable change in parallel with the increase in the incidence of IBD is diet. Intense urbanization and industrialization have led to a switch from more traditional regional diets, such as the Mediterranean, Japanese or Southeast Asian diets, to a western-style diet [69,70,71,72,73]. Persson et al. found an increased risk of both CD and UC associated with high calorie and sucrose intake and decreased risk associated with a high-fiber diet [74]. Experimental models proved that a high fat/high sugar western diet induces dysbiosis and alters host barrier function in the gut mucosa—key elements in the pathomechanisms of IBD [75]. However, the link between western-style diets and the homeostasis of gut microbiota is still under investigation. Nonetheless, it is increasingly evident that diet plays a key role in the pathophysiological mechanisms that lead to IBD. Table 3 details studies assessing the impact of various diets in patients diagnosed with IBD.

Md has been intensely studied due to the low incidence of IBD in areas of Europe where consumption of Md is common [60,61]. Md is also considered a possibly effective tool in IBD treatment due to its high potential to modulate gut inflammation [81]. A prospective study conducted by Chicco et al. on an Italian cohort of 142 patients diagnosed with IBD (84 with UC and 58 with CD) evaluated the short-term impact of adherence to Md during a span of 6 months based on quality of life (QoL) and disease activity [76]. The study concluded that QoL improved, while only 6.8% of patients still showed mild disease from a baseline of 23.7% in the UC group and 3.8% of CD patients showed mild disease from a baseline of 17%. These results were further confirmed by significant decreases in fecal calprotectin levels and normalization of CRP at the end of 12 months in both groups, countering a possible overestimation of the Md benefits caused by the large proportion of patients in clinical remission at the beginning of the study [76]. However, a recent cross-sectional study by Vrdoljak et al. observing a total of 94 IBD patients showed that patients had generally poor adherence to Md, more specifically lacking adherence to foods that were essential to the diet, such as fresh fruit and vegetables [77]. The large majority of the patients (86.2%) believed that a more controlled diet could be beneficial, thus underlining the importance of educational programs in the nutrition of IBD patients [77]. These findings are strongly supported by a large study conducted by Khalili et al. on 83,147 participants from Sweden which concluded that greater adherence to Md was inversely correlated with the risk of late-onset CD but not UC [78]. The importance of Md is further highlighted by findings in the studies by Chicco et al. and Vrdoljak et al. that showed an improvement in the lipid profile of patients adhering to the diet and, consequently, a possible benefit in terms of cardiovascular health [76,78].

A study conducted on 104 IBD patients by Gearry et al. showed improvement in overall symptoms in a majority of the subjects, especially with respect to bloating, flatulence, abdominal pain and diarrhea, when undergoing a low-FODMAP diet [21]. However, the study relied only on patient-reported data and could not monitor disease activity in relation to the Low-FODMAP Diet (LFD) [21]. A more recent randomized controlled trial by Bodini et al. assigned 55 IBD patients into two groups, one to follow a LFD and the other to follow a diet with a standard amount of FODMAPs. After a 6-week period, the patients in the LFD group showed statistically lower calprotectin levels compared to the control group and reported an increase in QoL as opposed to the standard FODMAP diet group [79]. However, a 2020 meta-analysis by Grammatikopoulou et al. found that results from randomised controlled studies (RCTs) on LFD are too heterogeneous and differences between studies regarding disease activity and symptom relief after adherence to LFD might be too great for the diet to be recommended in clinical practice [22]. Although the dietary restrictions required to adhere to an LFD might be a burden on patients with chronic illnesses and might negate any improvement in QoL, research has shown the beneficial impact of a low-FODMAP diet in patients suffering from IBD, relief in gastrointestinal symptoms being reported [82]. 

Other diets described in the literature that have been studied in relation to IBD are the Specific Carbohydrate Diet (SCD) and the Gluten-Free Diet (GFD) [80,83]. SCD, developed in the early 20th century as a treatment for celiac disease, supposedly reduces inflammation and restores microbiome homeostasis by eliminating sugars, cereal, milk and processed foods from the patient’s diet. Several small studies on pediatric populations of IBD patients reported symptom alleviation and a reduction in inflammatory markers [80,83,84]. Suskind et al. conducted a retrospective study on the impact of the SCD in pediatric IBD patients and found an improvement in the Pediatric Crohn’s Disease Score Index (PCDAI) as well as a reduction in inflammatory markers, such as CRP and fecal calprotectin, after 12 weeks of diet adherence [80]. These results are limited to a pediatric population, therefore further randomized controlled studies should be conducted in the future to assess disease activity and mucosal healing compared to regular diets and other restrictive diets in IBD [80]. When it comes to gluten-free diets (GFDs) and overall gluten intake, studies have attempted to assess the impact this type of diet has on the quality of life of patients with IBD [16,18,85]. Nevertheless, the lack of clinical trials makes recommending dietary therapies without gluten a complicated task. A study conducted by Herfarth et al. analysed the impact of GFD on 1647 patients diagnosed with IBD, concluding that more than half of the respondents reported improvement in symptoms over the course of the diet [18]. The results showed less bloating, nausea, abdominal pain, fatigue and diarrhea episodes, while adherence to the diet played an important role in the reduction of symptoms [18].

The recent expansion in our knowledge of the involvement of gut microbiota in inflammatory and autoimmune diseases has raised among researchers the question of the involvement of diet in disease pathogenesis and the potential benefits certain dietary changes could have in patients suffering from HLA-B27-associated pathologies. Table 4 and Table 5 present current existing nutritional guidelines and recommendations for patients suffering from PsA and IBD from the American College of Rheumatology (ACR) and the European Society for Clinical and Nutritional Metabolism (ESPEN) [85,86]. We also mention that, to our knowledge, we have not identified any existing guidelines or dietary recommendations in order to better manage patients with AS.

On one hand, the global trend of adopting a “western diet” has been strongly correlated in numerous studies with a predisposition to develop SpA or IBD and other diseases outside of the HLA-B27 spectrum. On the other hand, there is significantly less conclusive evidence concerning the direct benefits of restrictive diets regarding disease activity and overall quality of life. First, the restrictive nature of the diets can hinder long-term adherence and therefore reduce the benefits obtained from them. Secondly, the variability in the composition and duration of each diet makes it difficult to design the randomized controlled studies that are needed to obtain a more clear-cut perspective on the matter. Therefore, further research is needed to establish the therapeutic impact of dietary changes and education in HLA-B27-associated diseases.

Our review has several notable limitations that should be discussed. Firstly, the numbers of patients included in the studies considered were insufficient. Secondly, a precise assessment of the adherence to a specific diet is difficult. Thirdly, although uveitis has a high prevalence among HLA-B27-positive patients [8,9,10], we were unable to find any studies analysing the impact of diet in the management of this disease. Moreover, patients included in the studies follow different pharmacological therapies, thus representing methodological bias. Additionally, spondyloarthritis, psoriatic arthritis and inflammatory bowel disease have all been correlated with HLA-B27, although the percentage of positive patients varies among them, with most included studies not differentiating between HLA-B27-positive and -negative patients. This further limits the possibility of recommending a particular diet that will work for spondyloarthritis and other immune-mediated disorders. Nevertheless, although dietary recommendations are not to be made on the basis of HLA-B27 status, clinical management might improve if a tailored therapy and nutritional advice are applied.

## 5. Conclusions

Although significant evidence suggests that certain diets might improve patients’ symptoms, our study did not find one specific diet that is beneficial as a non-pharmacological therapy against spondyloarthritis and related immune-mediated disorders. The variability in the composition and duration of each diet, together with its restrictive nature, can hinder long-term adherence and, therefore, reduce the benefits obtained, proving the necessity of having a multidisciplinary team to manage patients with these types of diseases.

## Figures and Tables

**Table 1:** Studies analysing the impact of different diets in patients with AS.

Author (Year)	Study Design and Participants’ Characteristics	Type of Diet	Variables Evaluated	Outcome
Ebringer et al. (1996) [15]	Longitudinal prospective; 36 patients with AS	Low-starch	IgA ESR	Both IgA and ESR have significantly decreased over the 9-month period of the study, with the majority of patients reporting a reduction in the severity of symptoms
Appelboom and Durez (1994) [38]	Longitudinal prospective; 25 patients with AS, reporting morning stiffness of over 30 min, inflammatory lower back pain, multiple joint swelling	Dairy-free	General wellbeing Pain severity Duration of morning stiffness Articular stiffness Changes in NSAID treatment Considering the continuation of the diet	18 out of 25 patients had a good compliance with the diet, with 17 patients reporting moderate or good efficacy; 8 out of 13 patients could discontinue their NSAID therapy, with 6 patients continuing the diet for over 2 years, without any additional medication
Sundström et al. (2011) [39]	Longitudinal prospective; 111 patients (84 males, 27 females) with AS	Various dietary habits	Dietary habits Physical activity Medication Gastrointestinal symptoms Disease activity and functional capacity	27% of the patients reported gastrointestinal symptoms after consuming dairy, fatty or flour-rich foods. Nevertheless, the study did not yield conclusive evidence of a relationship between diet and disease activity but rather a correlation with gastrointestinal symptoms in patients with AS
Haugen et al. (1991) [40]	Questionnaire-based; 41 patients with AS	Fasting	Pain Stiffness Joint-swelling	More than half of the respondents reported less pain, stiffness and reduced joint-swelling

ESR: erythrocyte sedimentation rate; NSAID: non-steroidal anti-inflammatory drug.

**Table 2:** Studies analysing the impact of different diets in patients with PsA.

Author (Year)	Study Design and Participants’ Characteristics	Type of Diet	Variables Evaluated	Outcome
Caso et al. (2020) [19]	Cross-sectional observational study; 211 patients with PsA: 131 females and 80 males	Mediterranean diet	Disease activity Metabolic parameters	Most patients had moderate and high adherence to the diet; low adherence was associated with higher PsA activity
Klingberg et al. (2019) [20]	Interventional study; 41 patients with PsA	Low-energy diet Energy-restricted diet	Disease activity Weight loss	Significant reduction of disease activity after 6 months of diet Higher weight loss was associated with improvement in a dose–response manner
Di Minno et al. (2013) [49]	Interventional study; 126 overweight patients with PsA	Hypocaloric diet Free-managed diet	Minimal Disease Activity Metabolic parameters	74 patients managed a ≥5% weight loss, this being a predictor of minimal disease activity Regardless of diet, weight loss was crucial in predicting disease activity
Kharaeva et al. (2009) [50]	Case–control; 30 patients with PsA	Selenium aspartate, coenzyme Q10, vitamin E	Disease activity Pro/antioxidant balance in granulocytes, plasma Involved epidermis RBC sedimentation rate	Diet was effective in reducing oxidative stress in patients with PsA, together with improvement of clinical condition
Kristensen et al. (2018) [51]	Randomized controlled trial; 133 patients with PsA	Fish oil supplementation	Disease activity Use of analgesics Leukotriene formation from activated granulocytes	Decreased activity score for patients receiving the diet, decreased use of NSAID and paracetamol, reduced formation of leukotriene from activated granulocytes
Adawi et al. (2019) [52]	Cohort study; 37 patients with PsA	Ramadan practice	Disease activity Body mass index	Diet had a beneficial impact on disease activity, although weight loss did not vary between groups

**Table 3:** Studies analysing the impact of different diets in patients with IBD.

Author (Year)	Study Design and Participants’ Characteristics	Type of Diet	Variables Evaluated	Outcome
Herfarth et al. (2014) [18]	Cross-sectional observational study; 1647 IBD patients enrolled in the CCFA Partners internet-based cohort, 616 diagnosed with UC and 1031 with CD	Gluten-free diet	Symptom improvement Dietary adherence	65.6% of patients who have followed or are still on a gluten-free diet reported improvement in at least 1 symptom. Adherence was only associated with a reduction in fatigue, not any other symptoms
Gearry et al. (2008) [21]	Longitudinal retrospective; 72 IBD patients, 20 diagnosed with UC and 52 with CD	Low-FODMAP diet	Dietary adherence Change in gastrointestinal symptoms	Reduction of FODMAP intake is correlated with a reduction in abdominal pain, diarrhea, wind and bloating
Grammatikopoulou et al. (2020) [22]	Meta-analysis of randomized controlled trials; 205 IBD patients	Low-FODMAP diet	Disease activity Quality of life CRP and calprotectin levels	Studies showed conflicting results in terms of disease activity and quality of life. Most trials also found no significant difference in calprotectin or CRP levels after dietary restriction
Chicco et al. (2020) [76]	Interventional study; 142 IBD patients, 84 diagnosed with UC and 58 with CD	Mediterranean diet	Body mass index Fat body mass Lean body mass Disease activity Quality of life CRP and calprotectin levels Liver steatosis Serum lipid levels	Adherence to the Mediterranean diet showed an improvement in disease activity and reductions in inflammation markers and obesity-related parameters. Liver steatosis also showed notable improvement
Vrdoljak et al. (2020) [77]	Cross-sectional observational study; 94 IBD patients, 44 diagnosed with UC and 50 with CD	Mediterranean diet	Body mass index Waist circumference Disease activity Serum lipid levels hsCRP levels Dietary adherence Attitude regarding disease and eating habits	IBD patients adhering to the Mediterranean diet had higher HDL cholesterol levels. Although only 9 participants fulfilled the criteria for Mediterranean diet adherence, a majority of the patients considered that a more controlled diet would beneficially impact their symptoms
Khalili et al. (2019) [78]	Prospective cohort 83,147 participants enrolled in the Swedish Mammography Cohort and the Cohort of Swedish Men, respectively	Mediterranean diet	Dietary adherence Risk of CD and UC	Adherence to the Mediterranean diet is correlated with a lower risk of later-onset CD but not UC
Bodini et al. (2019) [79]	Randomized controlled trial; 55 IBD patients, 20 patients with UC, 35 patients with CD)	Low-FODMAP diet	Body mass index Disease activity CRP and calprotectin levels Quality of life	Although disease activity showed improvement in CD patients in the low FODMAP group, the UC cohort had similar scores after the 6-week dietary intervention regardless of diet. CRP levels showed no improvement; however, there was a significant reduction in calprotectin levels coupled with a modest increase in quality of life in the low-FODMAP group
Suskind et al. (2016) [80]	Cross-sectional observational study; 417 IBD patients, 221 diagnosed with UC and 196 with CD	Specific carbohydrate diet	Change in gastrointestinal symptomatology and association with laboratory values	Patients on the specific carbohydrate diet reported a decrease in abdominal pain, daily activity limitation, diarrhea and blood in the stools. However, less than half reported an improvement in laboratory values associated with the perceived remission

IBD: inflammatory bowel disease; UC: ulcerative colitis; CD: Crohn’s disease; CRP: C-reactive Protein; hsCRP: high-sensitivity CRP; FODMAP: fermentable oligosaccharides, disaccharides, monosaccharides and polyols.

**Table 4:** The 2018 American College of Rheumatology/National Psoriasis Foundation Guideline for the Treatment of Psoriatic Arthritis [85].

Disease	Diet	Recommendations
Psoriatic arthritis	Low-energy diet Hypocaloric diet Energy-restricted diet	Weight loss is recommended in patients with PsA for potential increase in pharmacological response

**Table 5:** European Society for Clinical and Nutritional Metabolism (ESPEN) nutritional recommendations for IBD patients [86].

Grade of Recommendation *		Recommendation
IBD	Strong consensus	A diet rich in fruit and vegetables, rich in n-3 fatty acids and low in n-6 fatty acids is associated with a decreased risk of developing Crohn’s disease or ulcerative colitis and is therefore recommended
	Strong consensus	There is no “IBD diet” that can be generally recommended to promote remission in IBD patients with active disease
	Strong consensus	No specific diet needs to be followed during remission phases of IBD
CD	Strong consensus	Exclusion diets cannot be recommended to achieve remission in active CD, even if the patient suffers from individual intolerances
	Strong consensus	Probiotic therapy should not be used for maintenance of remission in CD
UC	Strong consensus	Probiotic therapy should be considered for the maintenance of remission in ulcerative colitis

* Based on the Scottish Intercollegiate Guidelines Network (SIGN) methodology.
